# Tattoos in the general Swedish population: prevalence, determinants, and exposure characteristics

**DOI:** 10.1093/eurpub/ckaf178

**Published:** 2025-11-14

**Authors:** Christel Nielsen, Anna S Jöud

**Affiliations:** Department of Laboratory Medicine, Division of Occupational and Environmental Medicine, Lund University, Lund, Sweden; Department of Public Health, Clinical Pharmacology, Pharmacy and Environmental Medicine, University of Southern Denmark, Odense, Denmark; Department of Laboratory Medicine, Division of Occupational and Environmental Medicine, Lund University, Lund, Sweden; Department of Research and Education, Skåne University Hospital, Lund, Sweden

## Abstract

Tattoo ink on the European market has been reported to contain many human toxicants, which has raised concerns about health effects. Clarifying the exposure distribution in the population is key to understanding potential public health implications. We investigated the prevalence of tattoos in a population-based Swedish cohort of 13 046 individuals aged 20–68 years with exposure data collected through a questionnaire in 2021. In addition, we characterized the tattooed population in terms of sociodemographic and socioeconomic determinants and exposure characteristics using multivariable logistic regression, regressing tattoo status on sex, age, educational attainment, marital status, smoking, snuff use, and alcohol consumption. The age-standardized tattoo prevalence was estimated at 29% (95% confidence interval [CI] 27%–30%). Tattoos were more common in the younger age groups, particularly among females. Males were younger when they received their first tattoo and tended to have a larger tattooed body surface. Tattoo exposure was associated with unfavourable socioeconomic characteristics, with the highest odds ratio observed for current smokers compared with never-smokers (2.86; 95% CI 2.42–3.38). Our results suggest that any tattoo-related adverse effects may exacerbate existing inequalities in health. Additionally, the high prevalence in younger age groups indicates a likely shift in the characteristics of the tattooed population over time, which may have further implications for public health.

## Introduction

Tattooing is nowadays a mainstream mode of self-expression. There are numerous incentives for receiving a decorative tattoo, including body embellishment, self-affirmation, and marking achieved milestones [1–3]. New application areas have emerged, and tattooing is also used for cosmetic and medical purposes (e.g. nipple-areola complex reconstruction or to cover skin burns).

The commonness of tattoos has brought attention to potential health effects. The contents of tattoo ink have been under scrutiny during the last decade. Several reports show that inks on the European market often contain human toxicants, including polycyclic aromatic hydrocarbons, primary aromatic amines, and heavy metals [4–7].

To understand the public health implications of tattoos, the first step is to clarify the exposure distribution in the population. Despite an abundance of empirical data suggesting a rapidly increasing tattoo trend since the 1990s, scientific evidence of tattoo exposure in unselected groups is harder to come by. In studies of general populations with exposure data collected from 2015, the tattoo prevalence in Western Europe is reported to be 13%–21% [8–11], whereas the US prevalence is above 30% [1, 3]. In the 2015 version of the Swedish National Environmental Health Survey, the Swedish prevalence of permanent tattoos was estimated at 17% [12]. However, the current tattoo prevalence in the Swedish population is unknown.

With an increasing prevalence of tattoos, there will be a parallel increased demand for tattoo removal. Laser therapy is superior to other methods in terms of efficacy and safety, and it is therefore the mainstay of tattoo removal [13]. It works by photomechanical destruction of pigment agglomerates and photothermal degradation of pigments into smaller molecules that can be removed through lymphatic drainage. However, the degradation products are often more reactive than the original pigments [14], and laser therapy may produce immunological reactions such as hypersensitivity, allergy, and anaphylaxis [15]. It is therefore relevant to clarify the use of laser therapy for tattoo removal in the population.

Here, we investigate the prevalence of tattoo exposure in the general Swedish population and assess the determinants of tattooing as well as exposure characteristics, including the use of laser therapy for tattoo removal.

## Methods

### Setting

We used the population-based Swedish Tattoo and Body Modifications Cohort (TABOO) [16] that was established by pooling the age- and sex-matched controls from case–control studies investigating the association between tattoo exposure and cancer.

The study was approved by the Swedish Ethical Review Authority (no. 2019-03138), and all participants provided informed consent.

### Participants

The study population consisted of a random sample of 26 751 individuals aged 20–68 years identified in 2019 from the Total Population Register by Statistics Sweden, the national authority for official statistics.

### Questionnaire

The data collection procedure has been described in detail elsewhere [16]. In brief, exposure data were collected using a structured questionnaire administered by Statistics Sweden between February and April 2021. We used a generic title for the survey (i.e. *The role of new lifestyle factors in skin cancer, lymphoma, and other diseases*) to avoid conditioning participation on exposure status and reduce the risk of selection bias. Participants received the questionnaire by postal mail and could respond digitally or on paper. Three reminders were made.

Tattoos were defined as permanent body art obtained for decorative, cosmetic (i.e. permanent make-up, semi-permanent make-up, and microblading), or medical purposes. The participants were explicitly asked to also consider removed tattoos.

Crude estimates of the magnitude of exposure were obtained by asking participants to assess the total area of their tattooed body surface in hand palms (<1, 1–5, or >5), which is emerging as a consensus approach in tattoo research [8, 11, 16]. In addition, we asked about the number of tattoos, where motives were considered separate if ≥20 cm apart and cover-ups were regarded as separate tattoos, and the number of sessions. We assessed the age at first tattoo, ink colours, anatomical placement of tattoos, by whom and where tattoos were made, and the geographical region of tattooing. The questionnaire also covered tobacco use, alcohol consumption, and changes in sun behaviour after tattooing.

We asked participants if they had ever undergone laser therapy for tattoo removal. Those who answered yes received additional questions about the anatomical location, the number of treatments, and where the treatment was performed.

Annual data on educational attainment, marital status, and household disposable income were added from the Longitudinal integrated database for health insurance and labour market studies (LISA, Statistics Sweden).

### Statistics

To assess potential response bias, Statistics Sweden performed a drop-out analysis by comparing the sociodemographic characteristics of the responders and non-responders. Individual-level data on the non-participants were not available.

We calculated the age-standardized prevalence of tattoo exposure using direct standardization with the composition of the Swedish population in 2019 as the standard population.

We evaluated group differences using the Mann–Whitney *U* test for interval variables and the chi-square test for categorical variables. Observations with missing data were excluded from group comparisons.

We defined tattoo exposure as ever having a decorative, cosmetic, or medical tattoo. Univariable and multivariable logistic regression analyses were performed to estimate odds ratios (OR) of tattoo exposure in relation to potential sociodemographic and socioeconomic determinants. In the multivariable model, we modelled exposure status as a function of sex (female, male), age (continuous), educational attainment (primary and lower secondary, upper secondary, or post-secondary), marital status (married, unmarried, divorced, or widowed), smoking (current daily smoker, current occasional smoker, previous smoker, or never smoker), snuff use (current daily user, current occasional user, previous user, or never user), and alcohol consumption (almost daily, 4–6 days per week, 2–3 days per week, 1 day per week, 2–3 times per month, rare occasions, or never). We did not include household disposable income in the model as we were interested in individual-level determinants.

The statistical analyses were performed in SAS version 9.4 (SAS Institute, Cary, NC).

## Results

### Participants

The final sample consisted of 13 046 individuals, which corresponds to an overall participation rate of 49%. The participation rate was slightly higher in females, 53% compared with 45% in males. The results of the drop-out analysis have been published elsewhere [16]. In summary, the participation rates were higher in the older age groups, among married individuals, in those born in Sweden, and among individuals with higher educational attainment.

Of the participants, 56% were female, and the median age was 56 years (Table 1). Females had higher educational attainment than males, but males had slightly higher disposable household income. Although over 50% of the participants were married, a slightly higher proportion of males were unmarried, and a somewhat higher proportion of females were divorced.

**Table 1.** Characteristics and exposure status of the 13 046 participants stratified by sex

	Females (*n* = 7330)		Males (*n* = 5716)	
	*n* (%)	Median (Q1; Q3)^a^	*n* (%)	Median (Q1; Q3)
*Characteristic*				
Age		56 (49; 61)		57 (51; 61)
Educational attainment				
Primary and lower secondary	474 (6)		586 (10)	
Upper secondary	3034 (41)		2688 (47)	
Post-secondary	3815 (52)		2428 (42)	
Missing	7 (0.1)		14 (0.2)	
Household disposable income^a^ (×10^2^ SEK)		5618 (3425; 7802)		5839 (3677; 8061)
Missing	0		5 (0.1)	
Marital status				
Married	4075 (56)		3235 (57)	
Unmarried	1952 (27)		1690 (30)	
Divorced	1166 (16)		735 (13)	
Widowed	137 (2)		51 (0.9)	
Missing	0		5 (0.1)	
*Exposure status*				
Tattoo exposure, any	1627 (22)		1006 (18)	
Decorative tattoo	1464 (20)		982 (17)	
Cosmetic tattoo (ever)	211 (2.9)		23 (0.4)	
Medical tattoo	64 (0.9)		8 (0.1)	
*Tattoo exposure by age group*				
20–29	50 (50)		18 (27)	
30–39	204 (42)		80 (27)	
40–49	308 (30)		172 (26)	
50–59	622 (23)		357 (18)	
60–69	443 (15)		379 (14)	

a First quartile; third quartile.

### Prevalence

The crude tattoo prevalence was higher in females than in males, 22% compared with 18% (Table 1). Decorative tattoos were the most common, and 3% of females reported ever having a cosmetic tattoo. The proportion of males who had ever had a cosmetic tattoo was below 1%. Although medical tattoos were rare (<1%), they seemed to be more common in females than in males.

Table 1 shows the tattoo prevalence stratified by age and sex. Tattoos were more common in the younger age groups, and the prevalence decreased with increasing age. This pattern was particularly pronounced in females, where 50% of the participants aged 20–29 years had at least one tattoo. The difference in tattoo prevalence between females and males decreased with increasing age. The tattoo prevalence was almost twice as high in females as in males in the age group 20–29 years, whereas there was no evident sex difference in participants aged 60–69 years.

The age-standardized tattoo prevalence in Sweden was estimated at 29% (95% confidence interval [CI]: 27%–30%).

### Determinants

Tattooed participants were slightly younger than non-tattooed participants (Table 2). There were differences in educational attainment between tattooed and non-tattooed participants. This difference was observed for both sexes, but it was particularly pronounced in males. In males, the proportion of participants who had primary or lower secondary education was 16% among those with tattoos, compared with 9% in non-tattooed participants. The corresponding figures for females were 8% and 6%, respectively. Only 44% of tattooed females had post-secondary education, compared with 54% of non-tattooed females. The difference in educational attainment was even more pronounced in males; 25% of tattooed males had post-secondary education compared with 46% of non-tattooed males.

**Table 2.** Sex-stratified comparisons of characteristics of the tattooed and non-tattooed participants

	Females					Males				
	Tattooed (*n* = 1627)		Non-tattooed (*n* = 5703)			Tattooed (*n* = 1006)		Non-tattooed (*n* = 4710)		
	*n* (%)	Median (Q1; Q3)^a^	*n* (%)	Median (Q1; Q3)	*P*-value^b^	*n* (%)	Median (Q1; Q3)	*n* (%)	Median (Q1; Q3)	*P*-value
Age	1627	53 (43; 58)	5703	56 (50; 61)	<.001	1006	55 (46; 60)	4710	57 (51; 61)	<.001
Educational attainment					<.001					<.001
Primary and lower secondary	126 (8)		348 (6)			158 (16)		428 (9)		
Upper secondary	789 (48)		2245 (39)			591 (59)		2097 (45)		
Post-secondary	710 (44)		3105 (54)			256 (25)		2172 (46)		
Missing	2 (0.1)		5 (0.1)			1 (0.1)		13 (0.3)		
Marital status										
Married	777 (48)		3298 (58)			523 (52)		2712 (58)		
Unmarried	504 (31)		1448 (25)			315 (31)		1375 (29)		
Divorced	317 (19)		849 (15)			163 (16)		572 (12)		
Widowed	29 (2)		108 (2)			4 (0.4)		47 (1)		
Missing	0		0			1 (0.1)		4 (0.1)		
Disposable income, household^c^ (×10^2^ SEK)		5098 (3068; 7122)		5747 (3553; 7994)	<.001		5149 (3324; 7315)		5964 (3774; 8205)	
Missing	0		0				0		0	
Smoking				<0.001						<.001
Yes, daily	206 (13)		373 (7)			94 (9)		227 (5)		
Yes, occasionally	98 (6)		167 (3)			79 (8)		201 (4)		
No, but previously	682 (42)		1764 (31)			435 (43)		1364 (29)		
No, never	636 (39)		3389 (59)			395 (39)		2905 (62)		
Missing	5 (0.3)		10 (0.2)			3 (0.3)		13 (0.3)		
Snuff use				<0.001						<.001
Yes, daily	198 (12)		214 (4)			384 (38)		863 (18)		
Yes, occasionally	40 (2)		51 (0.9)			33 (3)		94 (2)		
No, but previously	126 (8)		195 (3)			205 (20)		822 (17)		
No, never	1249 (77)		5192 (91)			379 (38)		2909 (62)		
Missing	14 (0.9)		51 (0.9)			5 (0.5)		22 (0.5)		
Alcohol consumption, frequency				<0.001						.28
Almost daily	17 (1)		65 (1)			29 (3)		111 (2)		
4–6 days per week	57 (4)		239 (4)			48 (5)		293 (6)		
2–3 days per week	356 (22)		1399 (25)			309 (31)		1392 (30)		
1 day per week	258 (16)		963 (17)			181 (18)		903 (19)		
2–3 times per month	298 (18)		971 (17)			156 (16)		721 (15)		
Rare occasions	502 (31)		1467 (26)			194 (19)		951 (20)		
Never	135 (8)		585 (10)			84 (8)		325 (7)		
Missing	4 (0.3)		14 (0.3)			5 (0.5)		14 (0.3)		

a First quartile; third quartile.

b According to the Mann–Whitney *U* test for the interval variable and the chi-square test for categorical variables.

c Salaries, social security benefits, and income from capital, less tax, and other negative transfers.

The proportion of tattooed females who were married was 48% (58% in non-tattooed females), whereas it was 52% in tattooed males (58% in non-tattooed males). Tattooed participants were slightly more likely to be divorced, and the disposable income of their household was lower in tattooed participants compared with non-tattooed participants.

Tobacco use was more common among tattooed participants, but we observed no differences in the frequency of alcohol consumption. The proportion who reported current smoking (daily as well as occasional) was twice as high among tattooed participants in both sexes, and there were also larger proportions of previous smokers among the tattooed participants. Only 39% of tattooed females stated that they were never-smokers, compared with 59% of non-tattooed females. The corresponding proportions in tattooed and non-tattooed males were 39% and 62%, respectively. Snuff use was generally more prevalent among males. However, there were distinct within-sex differences between tattooed and non-tattooed participants. For instance, 12% of tattooed females reported current snuff use, whereas the corresponding figure in non-tattooed females was 4%. Among males, 38% of the tattooed participants reported daily snuff use, and the corresponding proportion in non-tattooed males was 18%.

In the multivariable regression model, sex, age, educational attainment, marital status, smoking, and snuff use—but not alcohol consumption—were associated with tattoo status (Table 3). We found the highest OR for current tobacco use. Current daily smokers had an OR of tattoo exposure of 2.86 (95% CI 2.42–3.38) compared with never-smokers, and current daily snuff users had an OR of 2.70 (95% CI 2.36–3.09) relative to individuals who never used snuff. There was also a distinct association between educational attainment and the likelihood of having tattoos. In relation to participants with post-secondary education, participants with primary education had an OR of having tattoos of 2.41 (95% CI 2.04–2.85), and participants with upper secondary education had an OR of 1.89 (95% CI 1.71–2.09). The regression analysis confirmed the higher OR of tattoos in females compared with males (OR = 1.75, 95% CI 1.57–1.95) and a decreasing OR with increasing age. Finally, we observed increased odds of having tattoos among divorced participants (OR: 1.50, 95% CI 1.32–1.71).

**Table 3.** Determinants of tattoo exposure

	Tattooed (*n*)	Non-tattooed (*n*)	OR (95% CI^a^)
Sex			
Female	1606	5629	1.75 (1.57–1.95)
Male	994	4659	1.00
Age (years)	2600	10 288	0.95 (0.94–0.95)
Educational attainment			
Primary and lower secondary	278	755	2.41 (2.04–2.85)
Upper secondary	1363	4290	1.89 (1.71–2.09)
Post-secondary	959	5243	1.00
Marital status			
Unmarried	807	2791	0.90 (0.81–1.01)
Divorced	473	1400	1.50 (1.32–1.71)
Widowed	31	152	1.02 (0.68–1.54)
Married	1289	5945	1.00
Smoking			
Current, daily	294	586	2.86 (2.42–3.38)
Current, occasional	175	362	2.13 (1.73–2.62)
Previously	1109	3095	1.98 (1.78–2.20)
Never	1022	6245	1.00
Snuff use			
Current, daily	576	1073	2.70 (2.36–3.09)
Current, occasional	73	144	1.92 (1.40–2.62)
Previously	331	1015	1.79 (1.53–2.09)
Never	1620	8056	1.00
Alcohol consumption			
Almost daily	45	175	1.01 (0.69–1.54)
4–6 days per week	103	525	0.81 (0.62–1.07)
2–3 days per week	662	2776	1.07 (0.89–1.29)
1 day per week	436	1852	1.04 (0.86–1.26)
2–3 times per month	450	1680	1.14 (0.94–1.38)
Rare occasions	688	2391	1.11 (0.92–1.33)
Never	216	889	1.00

a Confidence interval.

### Exposure characteristics

Males were younger than females when they received their first tattoo (median age 23 and 30 years, respectively; Table 4), and they tended to have a larger tattooed body surface. Although an area of total tattooed body surface of less than one hand palm was most common in both sexes (60% of the females and 48% of the males), a larger proportion of males had larger tattoos. Among male participants, 13% had a tattooed body area larger than five hand palms, whereas the corresponding proportion in females was 7%.

**Table 4.** Exposure characteristics among the 1627 tattooed female and the 1006 tattooed male participants

	Females		Males		
	*n* (%)	Median (Q1; Q3)^a^	*n* (%)	Median (Q1; Q3)	*P*-value^b^
Median age in years at first tattoo		30 (20; 43)		23 (18; 36)	<.001
Missing	15 (0.9)		7 (0.7)		
Area of tattooed body surface					<.001
<1 hand palm	970 (60)		473 (47)		
1–5 hand palms	526 (32)		390 (39)		
>5 hand palms	119 (7)		132 (13)		
Missing	12 (0.7)		11 (1)		
Number of tattoos^c^					.02
1	759 (47)		445 (44)		
2–3	530 (33)		305 (30)		
4–5	182 (11)		119 (12)		
6–9	98 (6)		83 (8)		
≥10	48 (3)		47 (5)		
Missing	10 (0.6)		7 (0.7)		
Number of tattoo sessions					<.001
1	741 (46)		437 (43)		
2–3	528 (32)		290 (29)		
4–5	181 (11)		110 (11)		
6–9	100 (6)		87 (9)		
≥10	66 (4)		72 (7)		
Missing	11 (0.7)		10 (1)		
Colour scheme					<.001
Black^d^	688 (42)		326 (32)		
Colour	201 (12)		178 (18)		
Black and colour	732 (45)		496 (49)		
Missing	6 (0.4)		6 (0.6)		
Individual colours^e^					
Black	1377 (85)		806 (80)		.003
Grey	232 (14)		186 (18)		.004
Brown	143 (9)		83 (8)		.63
Red	507 (31)		466 (46)		<.001
Blue	363 (22)		366 (36)		<.001
Green	397 (24)		336 (33)		<.001
Yellow	282 (17)		346 (34)		<.001
White	230 (14)		183 (18)		.005
Purple	116 (7)		43 (4)		.003
Pink	160 (10)		33 (3)		<.001
Orange	100 (6)		69 (7)		.47
Turquoise	112 (7)		46 (5)		.02
Skin tone (injected colour)	18 (1)		8 (0.8)		.43
Other	5 (0.3)		9 (0.9)		.04
Anatomical site of tattoos^e^					
Head	168 (10)		8 (0.8)		<.001
Neck	128 (8)		38 (4)		<.001
Right upper arm, chest, upper back	611 (38)		644 (64)		<.001
Left upper arm, chest, upper back	638 (39)		573 (57)		<.001
Right lower arm, hand	306 (19)		224 (22)		.03
Left lower arm, hand	361 (22)		272 (27)		.005
Abdomen, groin, genitals, lower back, buttocks	319 (20)		56 (6)		<.001
Right thigh, knee	54 (3)		28 (3)		.44
Left thigh, knee	37 (2)		25 (2)		.73
Right lower leg, foot	357 (22)		114 (11)		<.001
Left lower leg, foot	236 (15)		90 (9)		<.001
By whom and where tattoo was made^e^					
Professional tattoo artist in tattoo studio	1338 (82)		856 (85)		.06
Professional tattoo artist at other facility	193 (12)		119 (12)		.98
Cosmetic tattoo artist in cosmetic tattoo studio, beauty salon, or plastic surgery clinic	183 (11)		3 (0.3)		<.001
Health care professional at hospital or other health care establishment	61 (4)		4 (0.4)		<.001
Other, irrespective of where	94 (6)		149 (15)		<.001
Geographical regions of tattooing^e^					
Sweden	1478 (91)		818 (81)		<.001
Nordic countries (not Sweden)	62 (4)		146 (15)		<.001
Rest of Europe (not Nordic countries)	115 (7)		103 (10)		.004
Asia	74 (5)		85 (8)		<.001
Oceania	11 (0.7)		6 (0.6)		.80
USA	41 (3)		18 (2)		.22
Other	36 (2)		23 (2)		.90
Laser removal of tattoo					.36
Yes	36 (2)		17 (2)		
No	1576 (97)		974 (97)		
Missing	15 (0.9)		15 (1)		
Sun exposure after receiving tattoo					<.001
More frequent	4 (0.3)		7 (0.7)		
Less frequent	98 (6)		40 (4)		
Same frequency, but protects tattooed skin	252 (15)		87 (9)		
Unchanged	1254 (77)		862 (86)		
Missing	19 (1)		10 (1)		

a First quartile; third quartile.

b According to the Mann–Whitney *U* test for the interval variable and the chi-square test for categorical variables.

c Motives were considered separate tattoos if ≥20 cm apart, and cover-ups were considered two separate tattoos.

d Black included both black and grey shades.

e Multiple answers were possible. The proportion of participants stating each answer is expressed relative to the total, and *P*-values refer to differences in the proportion who stated each answer in relation to those who did not.

The colour scheme differed between the sexes. Overall, it was most common with a colour scheme consisting of both black and colour, but 42% of females had only black tattoos. The corresponding proportion in males was 32%, and a higher proportion of males reported that they had only coloured tattoos (18% compared with 12% of females). Of individual ink colours, black, followed by red, was most common in both sexes.

The anatomical location of tattoos varied by sex. Males were most often tattooed on the upper arms, chest, and upper back. Although the same locations were most common also in females, larger proportions reported tattoos on their lower legs and feet, as well as on the abdomen, groin, genitals, lower back, and buttocks. Approximately 10% of females had tattoos on the head (including the facial area), whereas the corresponding prevalence in males was below 1%.

More than 80% of tattooed participants reported receiving tattoos from a professional tattooer in a studio setting. In females, 11% had been tattooed by a cosmetic tattooer in a studio, beauty salon, or plastic surgery clinic, whereas 4% had received a tattoo at a healthcare establishment. A larger proportion of males, 15% compared with 6% of females, had been tattooed by a non-professional.

Most tattoos, 91% in females and 81% in males, were received in Sweden. It was more common among males to get tattooed outside Sweden, particularly in other Nordic countries (15%) and in Europe (10%). Five percent of females and 8% of males had received a tattoo in Asia.

Irrespective of sex, 2% of participants had undergone laser treatment for tattoo removal. Most participants reported that their sun behaviour remained unchanged after receiving tattoos. A larger proportion of females than males, 15% and 9%, respectively, reported that their frequency of sun exposure was unchanged, but that they protect tattooed skin by covering it or by using sunscreen. Less than 1% reported increased sun exposure after tattooing.

## Discussion

The age-standardized tattoo prevalence in the cohort was 29% (95% CI 27%-30%), which agrees with the prevalences reported in other population-based studies published within the last 5-year period. The tattoo prevalence was estimated at 21% in Denmark in 2021 [11], at 18% in France in 2018–2019 [1], and at 35% in the USA in 2017 [3]. Our study and the studies from Denmark and the USA were performed within the framework of larger surveys with a general scope, and it was not evident to the participants that the purpose was to investigate tattoo exposure. Selective participation conditioned on exposure status is therefore unlikely, which suggests that the results are internally valid. In agreement with the literature, we did, however, observe distinct differences in prevalence between the sexes and different age groups [1, 3, 8–11]. This emphasizes the need for standardization of the prevalence of tattoos to achieve externally valid estimates of population exposure.

Although decorative tattoos were most common, 3% of females reported ever having a cosmetic tattoo (and 10% stated being tattooed on their head, including the facial area), whereas the proportion of males was less than 1%. Most previous research has approached tattoo exposures as one single entity, i.e. they have not specified the type of tattoo under study [1, 3, 8–11], and comparison is therefore not possible. However, the prevalence of cosmetic and medical tattoos in the study by Renzoni *et al.* [8] agrees with our findings. With an increasing number of application areas of cosmetic tattoos, e.g. scalp micropigmentation, and an anticipated increased demand for laser therapy for tattoo removal, it is important to follow time trends in both exposure prevalence and exposure characteristics to detect potential public health effects.

We found that tattoos were more common among individuals with lower socioeconomic status. This finding was consistent across variables: tattooed individuals had lower educational attainment, were more often smokers and snuff users, and had lower household disposable income. The importance of these determinants was confirmed in the multivariable regression model, with current smoking status showing the strongest association when compared to never-smokers [3, 17]. The higher smoking rate in tattooed individuals agrees with the literature [3, 10, 17, 18], where it has been interpreted as an indication of a higher inclination for risk-taking behaviours. However, we found no evidence that tattooed individuals would have a riskier alcohol consumption pattern, which has been reported previously [3, 17].

Interestingly, we observed a larger proportion of tattooed females than males with post-secondary education and a larger proportion of males than females with only primary education. These findings suggest that the distribution of tattoos over educational attainment levels may vary depending on sex, and future studies may explore sex-specific determinants of tattooing across sociocultural contexts.

In agreement with Morlock and Morlock [3], we found lower household disposable income among tattooed participants. This may partly be explained by a larger proportion of single households, as slightly higher proportions of tattooed participants were unmarried or divorced. The higher prevalence of tattoos in divorced participants could be interpreted as a reflection of the personal motivational factors for tattooing, i.e. to mark important life events [1, 8, 17].

In the context of sociodemographic differences, it is important to acknowledge that our study population was established from the age- and sex-matched controls in case–control studies of tattoo exposure and cancer. Thus, the study design resulted in a sample of mostly middle-aged participants (the median age was approximately 55 years at the time of the survey). We found substantially higher tattoo prevalences in the younger age groups, particularly in females, and the continuously increasing tattoo prevalence may result in a population shift towards more similar characteristics between tattooed and non-tattooed individuals. However, a younger study population than the TABOO cohort is needed to investigate whether this is the case, and such studies must be carefully designed to address the challenge of lower participation rates among younger cohorts.

The data suggested that there were distinct sex-specific patterns of exposure characteristics in the Swedish population. In general, men were younger when they received their first tattoo, had a larger tattooed body surface, and more frequently had colourful tattoos. These differences may be relevant from a public health perspective because they imply that, on the group level, males have more years of exposure (i.e. have longer exposure duration at a certain age) and are exposed to larger doses of tattoo ink and their degradation products compared with females. Taken together, the male exposure pattern may imply that males could be at an overall higher risk of adverse health effects, although this remains to be clarified in epidemiologic studies.

Some additional findings warrant consideration. Firstly, an unexpectedly high proportion of participants had been tattooed by a non-professional tattooer (15% of males and 6% of females). Stringent hygiene regulations apply to professional tattoo studios, but these are rarely fulfilled in the non-professional arena. Importers of tattoo ink from outside the European Union hold the responsibility for compliance of the imported ink with the concentration limits for chemical substances and the labelling requirements in entry 75 of Annex XVII of the REACH regulation, and for reporting the products to the Swedish Medical Product Agency’s register of tattoo inks on the Swedish market. However, non-professionals may buy tattoo ink directly from online marketplaces and thereby, knowingly or unknowingly, circumvent the stipulated responsibilities of importers. As such, the inks used by non-professionals can be assumed to be less controlled than those used by professional tattooers. For these reasons, the Swedish Medical Products Agency strongly advises against in-home tattooing [19]. In-home tattooing may also be associated with a larger dose of ink, as the amount of pigment needed to tattoo a square centimetre of skin depends on the skill level of the tattooer [20]. Considering these factors, it seems reasonable to assume that in-home tattooing could be associated with a worse health-risk profile than professional tattooing. Secondly, a non-negligible proportion of participants (8% of males and 5% of females) had received a tattoo in an Asian country where the tattoo ink market is less stringent and the risk of infection may be elevated due to insufficient tattoo-hygiene standards. Whether this has implications for public health remains to be clarified.

### Strengths and limitations

A strength was that our exposure assessment distinguished between decorative, cosmetic, and medical tattoos, and that it included laser therapy for tattoo removal. Our data, therefore, provide higher granularity compared with studies that have treated tattoo exposure as a dichotomous trait.

The participants were asked about several lifestyle-related exposures, and it was not evident that the specific focus of the data collection was to investigate tattoo exposure. As such, the estimated tattoo prevalence is likely a more valid representation of the true population prevalence compared with studies investigating selected groups.

A limitation was that we did not have access to individual-level data for non-responders, which limited our possibility to assess whether participation was related to exposure status. However, the overall prevalence of tattoos seemed reasonable when compared to the 2015 Swedish National Environmental Health Survey and the recently published literature from other Western countries.

## Conclusions

Tattooed individuals had less favourable socioeconomic characteristics, including a higher inclination to tobacco use (but not alcohol consumption) and lower educational attainment. Social inequalities shape both health outcomes and access to care, influencing perceived health, referral patterns, and treatment choices. Thus, there is a risk that adverse effects from tattoo exposure may exacerbate existing social disparities in health. In the current generation, males might be at higher risk of adverse health effects because of larger tattooed body surfaces, longer exposure duration at a certain age, and more colourful tattoos. However, because of the strong increase in tattoo exposure in younger age groups, particularly among females, exposure characteristics of the tattooed population are likely to shift. Therefore, continuous monitoring of both tattoo prevalence and potential health effects associated with exposure is warranted.

From a public health perspective, the high overall prevalence of tattoos implies that even a small increase in risk will have a substantial impact on the health of the population. Epidemiologic studies to clarify the health effects of tattoo exposure are therefore urgently needed.

## Data Availability

The data that support the findings of this study are available from lead investigator C.N. (christel.nielsen@med.lu.se), but restrictions apply to the availability of these data, which were used under license for the current study and so are not publicly available. Data are, however, available from the authors upon reasonable request and with permission of the Swedish Ethical Review Authority. Key points The age-standardized prevalence of tattoo exposure in Sweden was estimated at 29%. The tattoo prevalence was above 40% in females younger than 40 years. Tattoo exposure was associated with an adverse socioeconomic profile, particularly tobacco use. Any adverse health effects of tattoo exposure may exacerbate existing social inequalities in health. The high tattoo prevalence in the younger age groups indicates a likely shift in the sociodemographic characteristics of the tattooed population over time, which may have further implications for public health.
